# Surgical results and quality of life after subtotal petrosectomy

**DOI:** 10.1007/s00405-022-07443-2

**Published:** 2022-06-29

**Authors:** Simon Geerse, Rob J. de Haan, Fenna A. Ebbens, Maarten J. F. de Wolf, Erik van Spronsen

**Affiliations:** 1 Department of Otorhinolaryngology, Amsterdam University Medical Centers, Location AMC, Meibergdreef 9, 1105 AZ Amsterdam, The Netherlands; 2 Clinical Research Unit, Amsterdam UMC, University of Amsterdam, Amsterdam, The Netherlands

**Keywords:** Ear disease, Surgery, Mastoid, Surgery, Reoperation, Retrospective studies

## Abstract

**Purpose:**

Few data are available regarding subjective complaints and quality of life (QoL) after subtotal petrosectomy (STP). The purpose of our study was to assess long-term surgical results after STP, and to evaluate disease-specific, patient-reported outcomes including QoL and subjective hearing.

**Methods:**

A retrospective cohort study, including a postal survey, was performed in the Amsterdam University Medical Centers (Amsterdam UMC) location Academic Medical Centre (AMC). All patients who underwent a STP between 1990 and 2018 were included. Patient characteristics, indication for surgery, adverse events, disease recidivism, and patient-reported health outcomes were evaluated.

**Results:**

181 patients (183 ears) underwent a STP for several indications. The main indication was chronic otitis media (COM) with or without cholesteatoma (69%). In the total cohort, 8 residual cholesteatoma (5%) and 6 inclusion cholesteatoma 4% were detected. Postoperative (functional) health outcomes showed a significant negative impact on QoL in the STP cohort compared to normative data. Compared to patients without ear problems, the postoperative STP patients scored worse on almost all domains of the Chronic Ear Survey (CES) (*p* < 0.001). SF-36 scores of postoperative STP data showed negative *Z*-scores in almost all subscales, indicating lower levels of QoL compared to Dutch reference values. Most subscales of the Amsterdam Inventory for Auditory Disability and Handicap (AIADH) demonstrate a large-effect size in disadvantage of the STP cohort when compared to Dutch reference values.

**Conclusion:**

STP is a suitable technique to tackle severe ear disease. Despite its favourable surgical results, STP has a negative impact on several domains of patient’s QoL.

**Supplementary Information:**

The online version contains supplementary material available at 10.1007/s00405-022-07443-2.

## Introduction

Subtotal petrosectomy (STP) can be an effective solution for chronic or recurrent mastoid and/or middle ear disease, especially for ears with no functional hearing [1, 2]. In their review, Prasad et al. present an overview of the nomenclature and the several steps of the surgical technique [1]. In brief, it comprises a canal wall down mastoidectomy with complete exenteration of all air cell tracts which are involved in the disease or which are necessary to remove according to the indication of surgery. The Eustachian tube is obliterated and the external ear canal is closed into a blind sac. The cavity is obliterated with autologous abdominal fat soaked in antibiotic ear drops.

Over the last decades, several reports have been published on the effectiveness of STP for various indications [1, 3–5]. Prasad et al. presented a list of almost 20 different pathologies for which STP was performed [1]. Indications varied from chronic otitis media (COM) with cholesteatoma to temporal bone fractures and multiple kinds of tumours. STP is also often combined with the implantation of a hearing device [1, 6, 7]. Devices such as a cochlear implant (CI) or a vibrant sound bridge (VSB) are in most cases implanted in a single-stage procedure with favourable results [6, 7]. Because of the wide exposure of the middle/inner ear STP can offer technical advantages in cases of complex implantation [6]. Most of these studies focussed on outcome measures such as control of disease and surgical success rates. Research on postoperative complaints including quality of life (QoL) is scarce. Only Magliulo et al. interviewed 26 patients after STP. All patients suffered from a petrous bone fracture violating the otic capsule [3]. In this study, a combination of (partly validated) questionnaires previously proposed to evaluate postsurgical QoL in vestibular schwannoma patients was used [8–10]. Because of the lack of data on patient-reported outcome measures (PROMs) including QoL and subjective hearing after STP, the current study evaluated both objective surgical results as well as subjective patient-recorded outcomes.

## Materials and methods

### Participants

A retrospective cohort study, including a postal survey, was performed in the Amsterdam University Medical Centers (Amsterdam UMC), location Academic Medical Center (AMC), a tertiary otological referral center. All consecutively admitted patients, treated with a STP between 1990 and 2018, with a postoperative follow-up of at least 6 months were eligible for inclusion. Patients were excluded when clinical information was incomplete or the indication for surgery included malignant pathology. Patient characteristics in terms of sex, age, indication for surgery, simultaneous implantation of hearing devices, and time to follow-up (TFU) were collected. TFU was defined as the time between surgery and moment of last contact at our outpatient clinic. Adverse events occurring in the first 3 months postsurgery were evaluated and categorized according to the classification of Clavien et al. [11]. This classification (four grades) differentiates between: (I) non-life-threatening, no lasting disability, conservative treatment; (II) potentially life-threatening, residual disability, with or without revision surgery; (III) with residual disability, including organ resection or persistence of life-threatening conditions; and (IV) deaths as a result of complications. In addition, the outcome of postoperative Magnetic Resonance Imaging with Diffusion-Weighted Imaging (MRI-DWI) to detect residual or recurrent disease in obliterated cavities was evaluated. Audiometric evaluation was performed in those patients with both pre- and postoperative hearing tests available. All identified patients were informed and eligible to decline the use of their anonymised patient records. No rejections were received. The survey part of the study was approved by the Medical Ethical Committee of the Amsterdam UMC. Written informed consent was obtained from all participants.

### Survey: health outcome questionnaires

Three self-reported health outcome questionnaires were sent by post between June 2019 and June 2020 to all patients aged ≥ 18 years at the time of surgery. Patients were asked to complete the Dutch versions of the Chronic Ear Survey (CES), the Medical Outcome Study 36-Item Short-Form Health Survey (SF-36), and the Amsterdam Inventory for Auditory Disability and Handicap (AIADH) [12–14]. Patients were instructed to fill in the questionnaires based on their current health status and to return the questionnaires by post. The CES (APPENDIX 1) is a 13-item disease-specific questionnaire, aiming to measure health impact and treatment effectiveness in patients with COM. The questionnaire consists of three subscales: activity restriction (AR), symptoms (ST), and medical resource (MR). From the ST and MR subscales, two symptom specific selections were derived: otorrhea specific items (ST-2, ST-6, and MR-3) and hearing specific items (ST-1 and ST-5) [12]. Higher CES scores reflect less severe complaints. The SF-36 consists of 8 subscales representing physical (role) functioning, social functioning, emotional (role) functioning, vitality, body pain, and general health perceptions [13]. The scores are translated to a scale ranging from 0 to 100, with a higher score indicating better QoL. The physical and mental components of the eight subscales can also be combined into a physical component summary (PCS) and mental component summary (MCS) score [15]. The AIADH consists of 30 items subdivided into 6 subscales: intelligibility in quiet, intelligibility in noise, distinction of sounds, detection of sounds, auditory localization, and intolerance of noise. A higher score indicates better functional hearing. The postoperative CES scores were compared to previously published data on postoperative scores of patients treated for COM via a canal wall up mastoidectomy (CWUM) approach (*n* = 29) and a group of control patients (*n* = 23) who underwent eye surgery and had no ear complaints [12]. Postoperative subscale scores on the SF-36 and AIADH were compared to Dutch normative data [13, 16].

To rule out a possible bias due to the inclusion of patients operated > 5 years ago, a subgroup of patients operated in the last 5 years was evaluated separately. Results of this subgroup were compared to the results of the total cohort.

### Statistical analysis

Baseline characteristics, adverse events, and surgical results were summarized using simple descriptive statistics. Differences in D-CES (subscale and otorrhea and hearing specific) scores between the STP patients, CWUM patients, and controls were analyzed using the non-parametric Kruskal–Wallis test. When the Kruskal–Wallis test showed statistically significant score differences (*p* < 0.05) across the three groups, we performed post hoc pairwise comparisons, adjusting p values using Bonferroni correction for multiple testing. Differences between the SF-36 subscale scores of STP patients and Dutch reference values scores were expressed in standard Z-scores (difference between mean subscale score of the STP-group and mean subscale score of the Dutch reference group divided by the standard deviation (SD) of the reference group). Differences between the AIADH subscale scores of STP patients and Dutch reference values scores were expressed in Hedges' g effect size (difference between mean subscale score of the STP-group and mean subscale score of the Dutch reference group divided by the pooled SD). *Z* values and Hedges’ g values of ≥ 0.20 were considered as a small effect, ≥ 0.50 as a medium effect, and ≥ 0.80 as a large effect. [17]. All analyses were performed in SPSS 26.0 (Chicago, IL, USA) and STATA 15 (Texas, USA).

## Results

### Participants

Between 1990 and 2018, a STP was performed in 181 patients (183 ears). Of these 181 patients, 163 (164 ears) fulfilled the inclusion criteria. Eighteen patients (19 ears) were excluded [malignancy (*n* = 5), FUT < 6 months (*n* = 13)]. Baseline patient characteristics are presented in Table 1. Of the included patients, 16 patients were aged < 18 years and therefore only included for evaluation of surgical results. Chronic otitis media (COM) was the major indication for surgery (69%) followed by osteoradionecrosis (15%). The indication ‘miscellaneous’ includes diseases such as eosinophilic granuloma, Langerhans Cell Histiocytosis, and reactive processes with inconclusive histologic results. Median TFU was 59 months (range 6–304 months). A total of 123 patients (75%) had a TFU of > 2 years and 82 patients (50%) of > 5 years. In 140 patients (86%), follow-up at the outpatient clinic was combined with MRI-DWI.

**Table 1:** Baseline characteristics (*n* = 163)

Characteristics	Number of patients
Female-to-male ratio	72 (44%):91 (56%)
Age in years, median (range)	49 (6–85)
Indication for surgery	
Chronic otitis media	112 (69%)
Osteoradionecrosis	24 (15%)
Oto-liquorrhea	4 (2%)
Foreign body	3 (2%)
Miscellaneous	20 (12%)
TFU in months, median (range)	59 (6–304)

### Surgical results

Surgery was performed by four different surgeons. Single-stage implantation of a cochlear implant (CI) or a vibrant sound bridge (VSB) was performed in 12 and 4 patients, respectively. No implant failures occurred. In 52 patients, an adverse event occurred (32% of the total cohort). Most events were related to wound healing (*n* = 33, 63% of the adverse events) (Table 2). Revision surgery was needed in 14 patients (22% of the adverse events). A total of 7 patients (14% of the adverse events) had a Grade 3 adverse event (facial nerve paresis/palsy or cochlear hearing loss). Adverse events involving the facial nerve were not related to one specific indication for surgery [osteoradionecrosis (*n* = 3) and COM (*n* = 3)]. In 9% (*n* = 14) of all cases, MRI-DWI follow-up demonstrated signs of cholesteatoma in the obliterated middle ear or mastoid. In 8 cases, residual cholesteatoma was detected; in 6 cases, MRI-DWI revealed inclusion cholesteatoma. Of these cases, the median time to detection was 46 months postsurgery (range 3–92 months). In six cases with recidivism (residual or inclusion) of cholesteatoma, revision surgery was needed. In the other cases, a wait-and-scan policy was conducted. No recidivism of disease other than cholesteatoma was found during TFU. After excluding 26 patients with missing audiometric data, a total of 137 patients remained for evaluation of perioperative hearing. Preoperatively, 99 patients (72%) had a Pure Tone Average (PTA) of ≥ 80 dB HL. Postoperatively, another 19 patients (14%) had shifted to a hearing of ≥ 80 dB HL. All patients had a maximal air–bone gap (± 60 dB HL) after surgery.

**Table 2:** Adverse events (*n* = 52) using the Clavien classification

Grade	Complication	Number of patients	Treatment
I	Mild wound-healing problems	16	Conservative
	Abdominal hematoma	6	Conservative
	Prolonged admission because of abdominal drain production	2	Conservative
	Persistent liquorrhea	4	Conservative
	Necrosis of skin flap donor site	2	Conservative
	Decubitus of contralateral earlobe	2	Conservative
	Defect to temporomandibular joint	1	Conservative
	Facial paresis	1	Conservative
II	Moderate wound-healing problems	4	Surgery
	Fat plasty necrosis	2	Surgery
	Retroauricular skin fistula with protrusion of cholesteatoma	1	Surgery
	Epidermoid cyst in blindsac	1	Surgery
	Postoperative floppy earlobe	2	Surgery
	Infected skull implant	1	Surgery
III	Facial paresis	3	Conservative
	Facial paresis	2	Surgery
	Facial palsy	1	Surgery
	Iatrogenic damage to the cochlea	1	Conservative
IV	–	0	–

### Survey: health outcome questionnaires

Of the included 163 patients, 20 patients (12%) were deceased during follow-up. Death was unrelated to the indication for surgery or the operation itself. As 16 children (aged < 18 years) were not included in the survey part of the study, only 127 patients were eligible to complete the questionnaires. A response rate of 50% (64 patients) was achieved. In the subgroup of patients operated in the last 5 years, the response rate was 61% (22 out of 36 patients). The Kruskal–Wallis test showed significant differences (*p* values < 0.01) in D-CES (subscale as well as otorrhea and hearing specific) scores between the postoperative STP patients, postoperative CWUM patients, and control group. In Table 3, the post hoc comparisons between the three groups are presented. Compared to the control group, the postoperative STP patients scored significantly worse on all domains except for the need for medical resources and otorrhea. Postoperative CWUM patients had significantly more otorrhea and needed more medical resources compared to STP patients. SF-36 scores of postoperative STP data showed negative Z-scores in almost all subscales, indicating lower levels of QoL compared to Dutch reference values (Table 4). Most impairments were observed in physical (role) functioning and general health perceptions, with medium-effect sizes ranging from 0.54 to 0.56. Aggregating the 8 subscale scores to the PCS and MCS scores revealed an almost medium-effect size (*z* = − 0.43) in the physical domain and a small effect (*z* = − 0.14) in the mental domain. In Table 5, the AIADH scores compared to Dutch reference values are presented. Most subscales demonstrate a large-effect size in disadvantage of the STP cohort. Only the subscale ‘intolerance of noise’ showed a medium effect size (Hedges’ *g* = − 0.54).

**Table 3:** CES scores of postoperative STP patients compared to CES scores of postoperative CWUM patients and postoperative eye surgery patients (control group)

	Post-STP (*n* = 64)		Post-CWUM surgery (*n* = 29)		Post-STP (*n* = 64)		Control group (*n* = 23)	
	Median	IQR	Median	IQR	Median	IQR	Median	IQR
Activity restriction	13.0	5.0	12.5	6.0	13.0	5.0	16.0	0.0
	*p* = 0.75*				*p* < 0.001			
Symptoms	32.5	4.0	31.0	10.3	32.5	4.0	40.0	3.0
	*p* = 0.88				*p* < 0.001			
Medical resource	15.0	1.0	13.0	3.5	15.0	1.0	15.0	0.0
	*p* < 0.001				*p* = 0.45			
Total of the three domains	60.5	7.8	56.0	18.0	60.5	7.8	71.0	3.0
	*p* = 0.32				*p* < 0.001			
Otorrhea-specific items (ST-2, ST-6, MR-3)	16.0	0.0	16.0	6.0	16.0	0.0	16.0	0.0
	*p *= 0.001				*p* = 1.00			
Hearing-specific items (ST-1, ST-5)	6.0	4.0	5.0	5.5	6.0	4.0	12.0	0.0
	*p* = 1.00				*p* < 0.001			

Post hoc comparisons after a significant Kruskal–Wallis test

*IQR* interquartile range

*p values were adjusted by the Bonferroni correction for multiple testing. Higher median scores reflect lesser complaints

**Table 4:** SF-36 scores of postoperative STP patients (*n* = 64) compared to normative data

	Mean	SD	*Z-*score^a^
Physical functioning	71.6	30.3	− 0.56
Role physical	57.5	44.8	− 0.54
Bodily pain	74.9	25.2	− 0.02
General health perceptions	60.0	25.7	− 0.56
Vitality	64.6	21.9	− 0.23
Social functioning	74.4	25.5	− 0.46
Role emotional	72.6	40.3	− 0.31
Mental health	77.4	17.9	+ 0.02
Physical component Summary (PCS)	45.7	11.2	− 0.43
Mental component summary (MCS)	48.6	10.4	− 0.14

Interpretation *Z-*scores: 0.20: small-effect size; 0.50: medium-effect size; 0.80: large-effect size

^a^Differences between SF-36 scores of STP patients and Dutch reference values expressed in standard (*Z*) scores

**Table 5:** AIADH scores of postoperative STP patients (*n* = 59) compared to normative data

	Mean	SD	Hedges’ *g*^a^
Intelligibility in quiet	6.6	3.7	− 2.38
Intelligibility in noise	5.3	4.2	− 1.61
Distinction of sounds	13.6	5.1	− 3.71
Detection of sounds	7.8	2.1	− 5.15
Auditory localization	6.6	2.2	− 3.99
Intolerance of noise	1.2	1.1	− 0.54

Interpretation Hedges’ *g*: 0.20: small-effect size; 0.50: medium-effect size; 0.80: large-effect size

^a^Differences between AIADH scores of STP patients and Dutch reference values expressed in Hedges’ *g*

Repeated analysis of the subgroup of STP patients treated within the last 5 years demonstrated similar results (data not presented, but available on request and numbered as Tables 6, 7 and 8).

**Table 6:** Subgroup of recently operated patients (≤ 5 years)

	Post-STP (*n* = 22)		Post-CWUM surgery (*n* = 29)		Post-STP (*n* = 22)		Control group (*n* = 23)	
	Median	IQR	Median	IQR	Median	IQR	Median	IQR
Activity restriction	14.0	6.0	12.5	6.0	14.0	6.0	16.0	0.0
	*p* = 0.90*				*p* = 0.002			
Symptoms	32.5	7.0	31.0	10.3	32.5	7.0	40.0	3.0
	*p* = 1.00				*p* < 0.001			
Medical resource	15.0	1.0	13.0	3.5	15.0	1.0	15.0	0.0
	*p* = 0.005				*p* = 0.34			
Total of the three domains	60.5	10.8	56.0	18.0	60.5	10.8	71.0	3.0
	*p* = 0.87				*p* < 0.001			
Otorrhea-specific items (S2, S6, M3)	16.0	1.0	16.0	6.0	16.0	1.0	16.0	0.0
	*p* = 0.32				*p* = 0.54			
Hearing-specific items (S1, S5)	5.0	5.5	5.0	5.5	5.0	5.5	12.0	0.0
	*p* = 1.00				*p* < 0.001			

CES scores of postoperative STP patients compared to CES scores of postoperative CWUM patients and postoperative eye surgery patients (control group)

Post hoc comparisons after a significant Kruskal–Wallis test

Higher median scores reflect lesser complaints

*p values were adjusted by the Bonferroni correction for multiple testing

**Table 7:** Subgroup of recently operated patients (≤ 5 years)

	Mean	SD	*Z*-score^a^
Physical functioning	67.7	28.1	− 0.74
Role physical	51.2	47.1	− 0.72
Bodily pain	74.9	24.3	− 0.02
General health perceptions	57.2	26.3	− 0.69
Vitality	65.5	20.9	− 0.18
Social functioning	74.4	25.4	− 0.46
Role emotional	70.0	41.7	− 0.38
Mental health	75.9	18.2	− 0.07
Physical component summary (PCS)	45.4	9.8	− 0.46
Mental component summary (MCS)	48.1	11.4	− 0.19

Interpretation *Z*-scores: 0.20: small-effect size; 0.50: medium-effect size; 0.80: large-effect size

SF-36 scores of postoperative STP patients (*n* = 22) compared to normative data

^a^Differences between SF-36 scores of STP patients and Dutch reference values expressed in standard (*Z*) scores

**Table 8:** Subgroup of recently operated patients (≤ 5 years). AIADH scores of postoperative STP patients (*n* = 19) compared to normative data

	Mean	SD	Hedges’ *g*^a^
Intelligibility in quiet	6.1	4.2	− 2.83
Intelligibility in noise	5.2	4.6	− 2.10
Distinction of sounds	13.2	6.0	− 4.49
Detection of sounds	7.9	2.6	− 6.09
Auditory localization	6.5	2.9	− 4.48
Intolerance of noise	1.0	0.9	− 0.38

Interpretation Hedges’ *g: *0.20: small-effect size; 0.50: medium-effect size; 0.80: large-effect size

^a^Differences between AIADH scores of STP patients and Dutch reference values expressed in Hedges’ *g*

## Discussion

### Participants

In this study, a large cohort of STP patients suffering from benign pathology was evaluated, all with a long TFU. The used surgical technique was similar to the technique described by Prasad et al., except for the stapes suprastructure which was left intact in the current cohort [1]. In our opinion, removing the stapes suprastructure increases the risk of damaging the remaining perceptive hearing or vestibular function.

### Surgical results

The main adverse events observed in the current cohort were wound-healing problems (retroauricular, blindsac, and donor site). Lyutenski et al. evaluated their wound problems in three different closure techniques performing STP [18]. Their revision surgery rate was 12% due to wound problems and they did not find any significant differences between the different techniques. A previously published review of literature showed rates of wound problems between 3.5% and 35.6% [1]. The amount of wound-related adverse events (20%) and related need for revision surgery (5%) in the current cohort is comparable to this previously published data. Only a few grade 3 adverse events related to the facial nerve and cochlea were observed. These adverse events were not related to one specific indication for surgery. The review of literature of Prasad et al. showed a cholesteatoma recidivism rate ranging from 1.5 to 17% [1]. Our cholesteatoma recidivism rate is within this range.

### Survey: health outcome questionnaires

STP is often presented as an effective and safe technique to finally and definitely solve chronic ear problems [1, 19]. ENT physicians seem to be very satisfied with the results in case of solving infection, low recidivism rate of disease, and low implant failure. However, contentment of the doctor does not automatically mean that the patient is also satisfied with the postoperative situation. In the last decade, there is a growing interest in Patient-Recorded Outcome Measures (PROMs) [12, 20, 21]. PROMs add information regarding the impact of surgery and postoperative symptoms to QoL [22]. To our knowledge, the patients’ point of view regarding STP has never been studied in depth. Only Magliulo et al. evaluated QoL in 26 patients suffering from petrous bone fractures violating the otic capsule who underwent an STP [3]. In comparison to our study group, those patients possibly reflected more problems related to the recent trauma. In the study of Magliulo, only disease-specific items were comprised and no questions regarding perceived QoL in general. In our study, PROMs were only assessed postoperatively and in some cases even after a long TFU. This a potential weakness of the current study. No strong conclusions can be drawn regarding the impact of the STP versus the impact of the disease on general QoL. However, several individual responses of patients did us realize that a STP in itself has more impact on general QoL than earlier assumed. Prospective studies could add valuable data regarding the subjective impact of chronic disease versus the postoperative situation. At the present time, there are several publications about surveys which are developed to measure the subjective measures of the pre- and postoperative situation [12, 23, 24]. However, no research has been performed on pre- and postoperative STP-situation yet. Comparing the CES scores to normative data easily explainable differences appeared. Because of the blindsac-closure patients did not have complaints of otorrhea and there was no long-lasting need for medical resources. Surprisingly, there was a significant difference in the AR scale in disadvantage of the STP group. This difference is possibly explained by the fact that patients had a very long history of activity restriction prior to surgery and were afraid to change their way of living afterwards. Compared to the post CWUM group significant differences were found in the MR scale and the otorrhea specific items in favour of the post STP group which could be explained by the fact that the ear canal was closed. Comparison of AIADH scores to normative data showed very large differences which could be fully explained by the disadvantages of unilateral hearing which in most patients was already present preoperatively. No consistent data regarding postoperative hearing rehabilitation other than hearing implants were present. Therefore, these results could not be evaluated in the current study. The relative lower effect size of the subscale ‘intolerance of noise’ could be the result of the fact that contralateral hearing was not changed preoperatively and therefore should not be typically different compared to normative data. A weakness of this study is the relatively low return rate of surveys in the total cohort (50%). One could argue if the results are representative for the total cohort. We believe that in a cohort like this with patients who were operated over a period of 28 years of time, it is very difficult to even reach a high return rate. As results in the subgroup of patients treated with a STP within the last 5 years were comparable to the total cohort, we consider that the data of the total study cohort is representative for the STP population in general. We believe that future research should focus on a prospective cohort study with surveys before and after surgery to compare results with this current study.

## Conclusion

Subtotal petrosectomy is an effective solution for chronic ear disease and showed favourable surgical results. This study provides valuable data regarding the patients’ point of view after STP to the limited data in the literature. The positive outcomes of the CES compared to ear surgery keeping the ear canal intact contrast with the unfavourable outcomes regarding general QoL and subjective hearing.

## Supplementary Information

Below is the link to the electronic supplementary material.

Supplementary file1 (DOCX 15 KB)
