# Supplementary material for: Surgical results and quality of life after subtotal petrosectomy
Source: Eur Arch Otorhinolaryngol. 2022 Jun 29;280(1):61–8. doi: 10.1007/s00405-022-07443-2 (PMC9813054; doi:10.1007/s00405-022-07443-2)
Supplement: Supplementary file 1 — Supplementary file1 (DOCX 15 KB) [file 405_2022_7443_MOESM1_ESM.docx]

**APPENDIX 1** *The Chronic Ear Survey (CES)*

| **Activity Restriction Based-Subscale** | | | | | | |
| --- | --- | --- | --- | --- | --- | --- |
| A1 | Because of your ear problem, you don’t swim or shower without protecting your ear. | | | | | |
|  | *Definitely true* | *True* | *False* | *Definitely false* |  |  |
| A2 | At the present time, how severe a limitation is the necessity to keep water out of your ears? | | | | | |
|  | *Very severe* | *Severe* | *Moderate* | *Mild* | *Very mild* | *None* |
| A3 | In the past four weeks, has your ear problem interfered with your social activities with friends, family and groups? | | | | | |
|  | *All of the time* | *Most of the time* | *A good bit of the time* | *Some of the time* | *A little of the time* | *None of the time* |
| **Symptom scale** | | | | | | |
| S1 | Your hearing loss is: | | | | | |
|  | *Very severe* | *Severe* | *Moderate* | *Mild* | *Very mild* | *None* |
| S2 | Drainage from your ear is: | | | | | |
|  | *Very severe* | *Severe* | *Moderate* | *Mild* | *Very mild* | *None* |
| S3 | Pain from your ear is: | | | | | |
|  | *Very severe* | *Severe* | *Moderate* | *Mild* | *Very mild* | *None* |
| S4 | Odor from your ear is very bothersome to you and/or others: | | | | | |
|  | *Definitely true* | *True* | *Don’t know* | *False* | *Definitely false* |  |
| S5 | The hearing loss in your affected ear bothers you: | | | | | |
|  | *All of the time* | *Most of the time* | *A good bit of the time* | *Some of the time* | *A little of the time* | *None of the time* |
| S6 | In the past 6 months, please estimate the frequency that your affected ear has drained: | | | | | |
|  | *Constantly* | *5 or more times, but not constantly* | *3-4 times* | *1-2 times* | *Not at all* |  |
| S7 | The odor from your ear affected ear bothers you and/or others: | | | | | |
|  | *All of the time* | *Most of the time* | *A good bit of the time* | *Some of the time* | *A little of the time* | *None of the time* |
| **Medical Resource Subscale** | | | | | | |
| M1 | In the past 6 months, how many separate times have you visited your doctor, specifically about your ear problem? | | | | | |
|  | *More than 6 times* | *5-6 times* | *3-4 times* | *1-2 times* | *None* |  |
| M2 | In the past 6 months, how many separate times have you used oral antibiotics to treat your ear infection? | | | | | |
|  | *More than 6 times* | *5-6 times* | *3-4 times* | *1-2 times* | *None* |  |
| M3 | In the past 6 months, how many separate times have ear drops been necessary to treat your ear condition? | | | | | |
|  | *More than 6 times* | *5-6 times* | *3-4 times* | *1-2 times* | *None* |  |

Copyright © 1997 Massachusetts Eye and Ear Infirmary and Outcome Science, Lic.
